# Are paraplegic wheelchair users vulnerable to overuse syndromes?

**DOI:** 10.55730/1300-0144.6045

**Published:** 2025-05-30

**Authors:** Kutay TEZEL, Esra ÜLGEN KIRATLIOĞLU, Hüseyin KAYADİBİ, İlkay KARABAY, Mitat CEBECİ, Eda GÜRÇAY

**Affiliations:** 1 Department of Physical Medicine and Rehabilitation, Gaziler Physical Medicine and Rehabilitation Education and Research Hospital, Health Sciences University, Ankara, Turkiye; 2 Department of Biochemistry, Osmangazi University, Eskişehir, Turkiye

**Keywords:** Spinal cord injury, overuse syndrome, wheelchair, ultrasound

## Abstract

**Background/aim:**

The purposes of this study are to determine the frequency of upper extremity overuse syndromes in patients with spinal cord injury (SCI) using manual wheelchairs, to evaluate these syndromes clinically and sonographically, and to identify possible predisposing factors.

**Materials and methods:**

A total of 38 patients with traumatic SCI, aged over 18 years and using manual wheelchairs, were enrolled in this cross-sectional study. The patients were evaluated with clinical and sonographic findings of the wrist, elbow and shoulder joints. Functional capacity, physical ability and upper extremity symptoms, and quality of life were assessed using the functional independence measure, the short form of the disabilities of the arm, shoulder and hand questionnaire, and the 36-item short form (SF-36) health survey, respectively.

**Results:**

While the shoulder joint accounted for the majority of joint pain detected in 23 patients, bursitis was the most common sonographic pathology. As a result of clinical and sonographic evaluations, it was determined that 29 patients had overuse syndrome. The likelihood of developing overuse syndromes increased with longer daily wheelchair use (OR = 1.666; p = 0.048) and high lesion level (OR = 12.01; p = 0.052). It decreased with the SF-36 pain score (OR = 0.943; p = 0.027).

**Conclusion:**

The shoulder joint was the most commonly affected area in terms of pain, sonographic findings, and overuse syndrome. Prolonged daily wheelchair use, thoracic-level lesions, and lower SF-36 pain subscale scores might be determinants of the development of upper extremity overuse syndromes in paraplegic wheelchair users.

## 1. Introduction

Patients with spinal cord injury (SCI) have to use their upper extremities more frequently due to loss of function in their lower extremities. Approximately 40% of patients use a wheelchair for both mobilization and daily living activities [1]. Wheelchair use, pushing, and flexion and extension movements are frequently repeated beyond normal physiological angles. In addition, abnormal loads are placed on the upper extremity during push-ups and transfers [2]. Upper extremities are not biomechanically suited for repetitive activities and heavy load bearing. Overuse syndromes can occur as a result of excessive mechanical stress during wheelchair use and environmental conditions that are inappropriate for patients with SCI. Accordingly, the shoulder, elbow, and wrist joints are particularly affected in the upper extremities. This stress frequently causes problems such as tendinitis, tenosynovitis, tendinosis, tendon ruptures, and entrapped neuropathies [3,4]. Overuse pathologies restrict patients’ functional independence, activities of daily living, and even participation in rehabilitation programs. Risk factors such as advanced age, high body mass index, long-term injury, and prolonged daily wheelchair use may worsen this situation [5,6].

Acute effects of overuse can be reversed with appropriate rest and load reduction. However, patients with SCI cannot rest properly as they are dependent on mobilization and other activities of daily living [5]. Microinjuries that occur in the early period may trigger inflammatory and degenerative pathways in the future. Intraarticular pressure increases with mechanical stress and repetitive activities. Altered biomechanics and different stress distributions are associated with functional instability and pain in the upper extremity. Studies have shown that the frequency of wrist and hand pain is 30%–73%, elbow pain is 32%, and shoulder pain is 30%–73% in patients with SCI [2,7].

Overuse injuries can be prevented from becoming resistant or progressing through early detection and treatment. Although there are many specific diagnostic tests, ultrasonography (USG) has transformed the early assessment of these conditions. Its advantages include the absence of radiation exposure, low cost, easy accessibility, and high reliability in experienced hands. USG also enables both static and dynamic evaluations. It reduces the need for time-consuming and invasive examinations such as magnetic resonance imaging (MRI), computed tomography, radiography, and electroneuromyography (ENMG). Moreover, it minimizes the frequency of surgical interventions by accurately identifying pathologies that cause functional limitations and even loss of productivity [7–9].

Although wheelchair use is an important factor that may lead to upper extremity injury, studies on this subject are scarce. The main purposes of our study are to determine the frequency of upper extremity overuse syndromes in patients with SCI who use a manual wheelchair, to evaluate these pathologies both clinically and sonographically, and to identify possible prognostic or risk factors.

## 2. Materials and methods

### 2.1. Patients

A total of 38 patients with traumatic SCI, aged over 18 years and using manual wheelchairs, who were admitted to the inpatient or outpatient clinics of Gaziler Physical and Rehabilitation Medicine Training and Research Hospital between June 2020 and June 2023, were enrolled in this cross-sectional study.

Patients with SCI at or below the second thoracic level (Th2), classified as A, B, C, or D by the American Spinal Injury Association impairment scale (AIS), for at least 6 months and who had been using a manual wheelchair for more than 3 months were included. The manual wheelchairs were high-weight, low-performance models that required the user to forcefully move their arms and apply significant force for propulsion.

Subjects with a history of upper extremity injury, complex regional pain syndrome or surgery, cardiopulmonary problems, pressure sores that could be exacerbated by repeated transfers, or a body mass index (BMI) over 40 kg/m^2^ were excluded.

Of the 47 potentially eligible patients, four refused to participate, three had a BMI > 40 kg/m^2^, and two had a previous history of upper extremity trauma or surgery. Therefore, 38 patients completed the study.

### 2.2. Protocols and procedures

Demographic parameters such as age, sex, BMI, marital status, education level, etiology, duration of SCI, lesion level, AIS score, duration of daily wheelchair use, and the number of transfers and push-ups per day were recorded.

### 2.3. Outcome measures

Since there is no definitive diagnostic method for all upper extremity overuse syndromes, studies have generally relied on patients’ symptoms and physical examination findings [10–12]. In our study, sonographic evaluation was included as a sensitive diagnostic method to detect potential pathologies at an early stage.

The outcome measures regarding the wrist, elbow, and shoulder joints were evaluated using three criteria: symptoms, clinical findings, and sonographic findings. The presence of at least two criteria for each joint was considered indicative of overuse syndrome.

#### Symptoms

The presence of any one of the following symptoms was considered sufficient to meet one criterion: pain or numbness in the hand, or tingling in the first three fingers for carpal tunnel syndrome (CTS), and pain or tingling in the ring and small fingers for cubital tunnel syndrome (CuTS). The presence of pain in unilateral and/or bilateral joints was considered as a single finding per patient. The severity of pain in the wrist, elbow, or shoulder during rest or passive range of motion was measured using a 10 cm visual analog scale (VAS), in which a score of ≥ 4 was considered “painful”.

#### Clinical findings

Several provocative tests should be considered to aid in the evaluation and diagnosis of overuse syndromes. The presence of any one of the following findings was considered sufficient to meet one criterion: (1) Phalen’s test, Tinel’s test, or atrophy of the thenar muscles for CTS; (2) Tinel’s sign or atrophy of the intrinsic hand muscles for CuTS; (3) pain during extension or flexion of the wrist against resistance for lateral or medial epicondylitis. Clinical evaluation of the shoulder joint was classified into four groups of tests: Hawkins and Neer tests for subacromial bursitis and rotator cuff tendinitis (Group 1); Yergason and Speed tests for biceps tendinitis (Group 2); drop arm test for rotator cuff rupture (Group 3); and forced adduction test for acromioclavicular joint (ACJ) pathology (Group 4).

#### Sonographic findings

Musculoskeletal sonography was performed on all patients by a single physiatrist with 10 years of experience in musculoskeletal sonography, who was blinded to the study design and clinical data, using a 5–12 MHz linear-array transducer (Logic e portable; GE Healthcare, Wuxi, China). Scanning was performed while the subjects were in a sitting position. Each structure (soft tissue, joint, nerve) was scanned in both longitudinal and axial planes according to the criteria described by previous authors [13,14].

**Figure 1:**
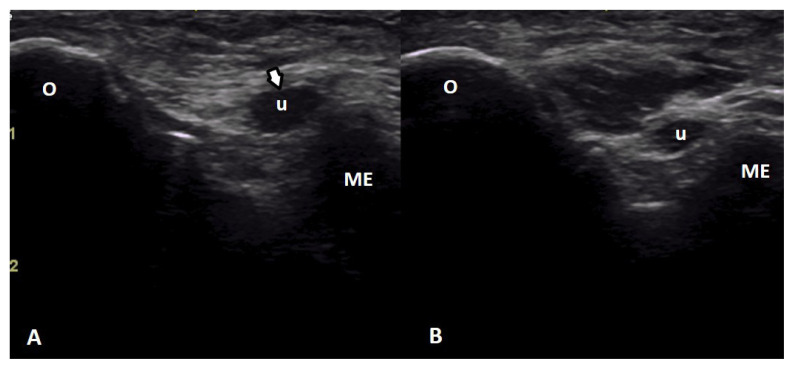
Cubital tunnel (CuT) syndrome. Axial US image at the CuT level reveals a swollen and hypoechoic ulnar nerve (u) with increased cross-sectional area (arrow) (A). Normal side (B). O: olecranon; ME: medial epicondyle; u: ulnar nerve.

**Figure 2:**
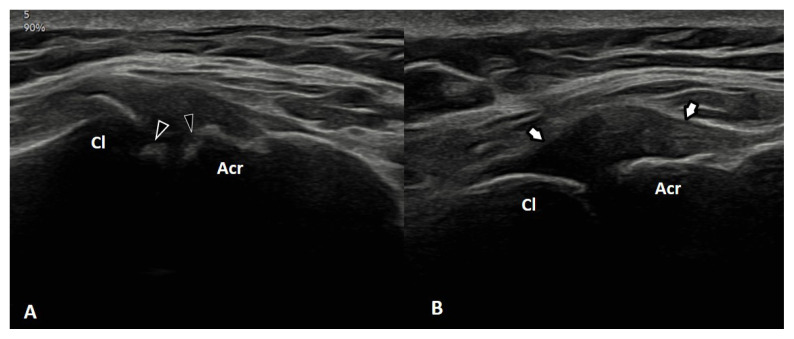
Acromioclavicular joint (ACJ) degeneration. Longitudinal US images over the ACJs reveal cortical irregularities at the joint surfaces (arrowheads) (A) and hypoechoic fluid collections distending the joint capsule (arrows) (B). Cl: clavicle; Acr: acromion.

For CTS diagnosis, the cut-off point for the cross-sectional area (CSA) of the median nerve is considered to be 9 mm^2^, and 10 mm^2^ for the ulnar nerve in CuTS [15,16]. Sonographic changes consistent with lateral or medial epicondylitis at the elbow; biceps tendinitis or tenosynovitis; rotator cuff tendinitis or rupture; subacromial bursitis; and ACJ degeneration at the shoulder were identified according to the literature [11,17]. Examples of different pathologies (cubital tunnel syndrome in Figure 1A and normal side in Figure 1B, ACJ pathological findings in Figures 2A and 2B) were presented.

The patients were evaluated with clinical and sonographic findings in terms of overuse syndromes of the wrist, elbow and shoulder joints on both sides.

The investigators assessed functional capacity in daily living activities using the Turkish version of the functional independence measure (FIM), including only the self-care and mobility subscales. A total of nine activities (six for self-care, three for mobility) in FIM are assessed for functional independence using a 7-point scale for each [18].

The short form of the disabilities of the arm, shoulder and hand questionnaire (Quick-DASH) was used for the assessment of physical ability and symptoms of the upper extremity [19]. Possible scores range from 0 to 100, with 0 indicating no difficulty or symptoms and 100 reflecting severe difficulty.

Health-related quality of life (QoL) was assessed using the Turkish version of the 36-item short form (SF-36) health survey. It was constructed to represent eight health concepts, including physical functioning, role-physical, bodily pain, general health, vitality, social functioning, role-emotional, and mental health [20].

### 2.4. Ethical considerations

The study was approved by the local ethics committee of a medical center under the number E2-21-931, and the study protocol was carried out in accordance with the Declaration of Helsinki. Subjects provided written informed consent prior to enrollment. Patients were informed that they were free to withdraw from the study at any time.

### 2.5. Statistical analyses

Data analyses were performed using SPSS (version 26; IBM Corp., Armonk, NY, USA). The normality of the distribution of continuous variables were assessed using the Shapiro–Wilk test. Continuous variables were presented as mean ± SD for normally distributed values and as median for nonnormally distributed ones. Independent samples t-test or Mann–Whitney U test was used to compare variables between groups as appropriate. Categorical variables were expressed as numbers and percentages, and compared by chi-square or Fisher’s exact test, as appropriate. An a priori power analysis was performed to determine the sample size using the G*Power program (Heinrich Heine University, Düsseldorf, Germany), with 80% power and an effect size of 0.79, assuming an α value of 0.05 [21,22]. Logistic regression analysis was used to identify the variables associated with overuse syndromes. For the multivariate logistic regression analysis, the possible factors identified by univariate analysis were entered into the model to determine predictors of the most common overuse syndromes. A p-value < 0.05 was considered statistically significant; however, a p-value < 0.100 was accepted as significant in univariate logistic regression analysis.

## 3. Results

Demographic and clinical characteristics of the patients are presented in Table 1. Thirty-eight patients with paraplegia at or below the Th2 level participated in this study. The number of females and males was equal. Of the subjects, 65.8% had injuries at the thoracic level, and 44.7% were classified as AIS A.

Clinical and sonographic outcomes are demonstrated in Table 2. The shoulder was identified as the most painful joint in the upper extremity (n = 10, 26.3%), and subacromial bursitis as the most common finding, present in 23 patients (60.5%). Pathological sonographic findings were detected more frequently than pain in each joint region of the upper extremity. Including overlapping pathologies, overuse syndromes were found in the shoulder joints of 22 patients, in the elbow joints of 13 patients, and in the wrist joint of eight patients.

In correlation analyses, when the presence of overuse syndromes was compared with clinical results, Quick-DASH scores increased in patients with CTS (r = 0.436, p = 0.006) and biceps tendinitis (r = 0.488, p = 0.002), while SF-36 pain subscale values decreased (CTS, r = −0.394, p = 0.014; biceps tendinitis, r = −0.428, p = 0.007). In addition, patients with CTS showed negative correlations with the FIM self-care subscale (r = −0.321, p = 0.05), and the SF-36 subscales of physical functioning (r = −0.426, p = 0.008), emotional (r = −0.531, p = 0.001), vitality (r = −0.472, p = 0.003), emotional health (r = −0.468, p = 0.003), and social functioning (r = −0.384, p = 0.017). There was no relationship between overuse syndromes in the elbow region in terms of functionality and QoL parameters.

Rotator cuff tendinitis/rupture and subacromial bursitis were the most common overuse syndromes in the patients’ upper extremities. According to multivariate analysis, the odds of developing CTS increased with hand pain (OR = 128.02, p = 0.023). Ulnar neuropathy was associated with CuTS (OR = 3.064, p = 0.025). Elbow pain and painful wrist extension during resistance were found to be independent predictors of lateral epicondylitis (OR = 64.63, p = 0.007; OR = 32.981, p = 0.035, respectively). The presence of medial epicondylitis increased by 21.33 times in the presence of pathological sonographic findings of medial epicondylitis (p = 0.025). Shoulder pain was a significant risk factor for the development of biceps tendinitis, rotator cuff tendinitis/rupture, and bursitis (OR = 25.008, p = 0.024; OR = 116.117, p = 0.002; OR = 3.62, p = 0.003, respectively). SF-36 physical functioning was inversely associated with biceps tendinitis (OR = 0.962, p = 0.043). Additionally, SF-36 emotional health for rotator cuff tendinitis/rupture (OR = 1.113, p = 0.024) and Group 1 tests for bursitis (OR = 73.53, p = 0.004) demonstrated positive associations. Multivariate logistic regression analysis revealed no potential risk factor for ACJ degeneration (Table 3).

Except nine patients, the rest had overuse syndromes based on clinical and sonographic evaluations. While the probability of developing overuse syndromes increased with daily wheelchair use (OR = 1.666, p = 0.048) and higher lesion level (OR = 12.01, p = 0.052), it decreased with higher SF-36 pain scores (OR = 0.943, p = 0.027) (Table 4).

## 4. Discussion

Patients with SCI who require a wheelchair frequently use their upper limbs for transfers, personal care, push-ups, and mobility. Therefore, the function and importance of the upper limbs have become evident, increasing the likelihood of overuse syndromes. It has been shown that 73% of these patients may experience some form of musculoskeletal pain during their lifetime [23].

This cross-sectional study showed that the shoulder joint was the most commonly involved region in terms of pain, pathologic sonographic findings, and the presence of overuse syndromes. Independent predictors of overuse syndromes in our study included the presence of pain (in the hand for CTS, in the elbow for lateral epicondylitis, and in the shoulder for biceps tendinitis, rotator cuff tendinitis, and bursitis); pain during resisted wrist extension for lateral epicondylitis; positive Hawkins and Neer tests for bursitis; pathologic sonographic findings for CuTS and medial epicondylitis; SF-36 emotional health scores for rotator cuff tendinitis; and SF-36 physical functioning scores for biceps tendinitis. Additionally, increased daily wheelchair use time, higher SCI level, and lower pain domain scores may be significant risk factors for developing overuse syndromes.

In SCI patients, the shoulder joint is the most vulnerable and painful area, with prevalence rates ranging between 30% and 73% [24,25]. The patient’s age, level of injury, and duration since injury were found to be correlated with shoulder pain [26,27]. In our study, consistent with many others, the shoulder joint was found to be the most affected area based on pain (26%), pathological sonographic findings (bursitis, 60.5%), and the presence of overuse syndromes (58%). The main reasons for this result may include muscular imbalance during transfers and pushing, increased intraarticular pressure exceeding arterial pressure by more than twofold, and the anatomical structure of the shoulder joint, which allows a wider range of motion than any other joint in the body [28,29].

In a clinical trial, the shoulder joints of 41 SCI wheelchair users with or without pain were screened by MRI, and the findings demonstrated no correlation between pain and pathology [30]. It was concluded that shoulder pathologies are equally present in persons with and without shoulder pain. In our study, USG was used as an imaging method, which differs from MRI but has been shown to correlate well with it in previous studies [31]. Similarly, pathological joint findings were observed more frequently than patient-reported pain. Furthermore, pain symptoms in all evaluated joints were associated with the development of overuse syndromes, except for CuTS and medial epicondylitis. Additionally, the pain subscore in QoL evaluation was a predictor of the occurrence of overuse syndromes. Based on the idea that clinical and radiological findings are not always synchronized in clinical practice, we aimed to determine a threshold level of association among the evaluation parameters to improve the diagnostic accuracy of overuse syndromes in our study. We consider that this approach may enhance diagnostic precision.

Elbow pain has been reported in 6%–32% of patients with SCI [32,33]. During transfers, push-ups and wheelchair use, the forearm is pronated and the elbow is in extension. Excessive loading and repetitive propulsive forces on the elbow joint and surrounding soft tissues may pose a risk, particularly for lateral epicondylitis. In this study, lateral epicondylitis was the most common overuse syndrome (15.8%) identified in the elbow joint. Pain in the elbow and pain during wrist extension were noted to be significant risk factors for lateral epicondylitis. The other overuse syndromes were CuTS and medial epicondylitis, with pathologic sonographic findings being the determining factors for both. Accordingly, when preventing upper extremity injuries, especially elbow pathologies, it may be appropriate to use the wheelchair within the limits of pain and to perform screening with sonography.

CTS has been identified as the most common cause (49%–73%) of hand and wrist pain in manual wheelchair users with SCI [34]. In light of the literature, older age, female sex, disease duration, and higher BMI have been reported as potential risk factors for CTS in the SCI population [12]. Although hand pain was the only factor associated with CTS in this study, correlation data revealed that CTS had negative effects on self-care, physical ability, and health-related parameters. Currently, we have not found any data reflecting the relationship between CTS and functionality or QoL in wheelchair users. The results of different studies are needed to provide more detailed comments on this subject.

When patients with and without overuse syndromes were compared, it was noted that increased daily wheelchair use time, higher SCI level, and lower pain subscores were significant risk factors for the occurrence of these syndromes. Since trunk stabilization is impaired in thoracic lesions due to involvement of the abdominal and erector spinae muscles, the force required during transfers and while pushing the wheelchair increases compared to lumbar lesions [35,36]. This situation may provoke imbalances between muscles in different planes of the upper extremity and may cause overuse syndromes to occur more frequently.

The limitations of this study were the small number of patients, the absence of a control group (e.g., SCI patients who ambulate or powered wheelchair users), the cross-sectional nature of the study, the unequal distribution of patients at the thoracic and lumbar levels, the lack of ENMG confirmation for entrapped neuropathies, and the exclusion of other possible upper extremity pathologies (e.g., radial nerve damage, triangular fibrocartilage complex damage, de Quervain’s tenosynovitis, ganglion cysts). The advantages of our study include the homogeneous distribution in terms of sex, the inclusion of both outpatients and inpatients, and the detailed and comprehensive evaluations accompanied by clinical and imaging methods.

The longer survival of patients with SCI has led to a new set of conditions grouped under the term “overuse syndrome”. This study revealed the shoulder joint as the most commonly involved region in terms of pain, pathologic sonographic findings and the presence of overuse syndromes. Sonographic evaluation showed that abnormal joint findings in the upper extremity were more prevalent than the pain reported by the patient. Moreover, increased daily wheelchair use time, thoracic-level lesions, and lower pain subscores might be predictors of developing overuse syndromes.

During the rehabilitation of patients with thoracic lesions who are long-term daily wheelchair users, it is essential to thoroughly screen the entire locomotor system, including unaffected areas. Clinical evaluations should be complemented with sonographic examinations to identify possible musculoskeletal pathologies, as patients may ignore other problems due to their existing major condition. However, underdiagnosed problems can hinder the rehabilitation process, render healthy upper limbs vulnerable to overuse syndromes, and eventually necessitate surgical intervention.

Therefore, in the early phase of treatment, focus may be placed on improving wheelchair usage skills, including push-ups, rest/sitting position and time, splint use, transfers, weight-bearing activities, and training to protect joint structures and restore muscle strength.

Finally, a follow-up design that includes larger patient groups can provide more accurate results by showing changes over time and lead to a more functional lifestyle for patients.

## Figures and Tables

**Table 1:** Demographic and clinical characteristics of the patients.

Variables		n (%), median (min–max)
**Age** (years)		31 (20–70)
**Sex**	Male	19 (50)
	Female	19 (50)
**BMI** (kg/m^2^)		23.29 (17.3–31.64)
**Marital status**	Married	21 (55.3)
	Single	17 (44.7)
**Education level**	Illiterate	1 (2.6)
	Middle school	16 (42.1)
	High school	7 (18.4)
	University	14 (36.8)
**Etiology**	Motor vehicle accident	18 (47.4)
	Violence	4 (10.5)
	Falls	8 (21.1)
	Others	8 (21.1)
**Time since injury (months)**		19.5 (6–120)
**Level of injury**	Thoracic	25 (65.8)
	Lumbar	13 (32.4)
**AIS**	A	17 (44.7)
	B	11 (28.9)
	C	8 (21.1)
	D	2 (5.3)
**Wheelchair use** *(hr/day)*		3 (1–10)
**Number of transfers** *(day)*		10 (4–35)
**Number of push-ups** *(day)*		20 (0–60)

BMI: Body mass index; AIS: American Spinal Injury Association impairment scale

**Table 2:** Clinical and sonographic outcomes of the patients.

Variables	n (%), median (min–max)
**Pain**	
Hand	7 (18.4)
Elbow	6 (15.8)
Shoulder	10 (26.3)
**QDASH**	8.25 (0–36.3)
**FIM**	
Selfcare	34 (17–42)
Mobility	18 (5–21)
**SF-36**	
Physical functioning	17.5 (0–35)
Role physical	75 (0–100)
Role emotional	100 (0–100)
Vitality	55 (25–90)
Emotional health	76 (28–100)
Social functioning	75 (0–100)
Pain	77.5 (22.5–100)
General health	55 (25–80)
**Sonographic outcomes**	
Median neuropathy	10 (26.3)
Ulnar neuropathy	5 (13.2)
Lateral epicondylitis	8 (21)
Medial epicondylitis	5 (13.1)
Biceps tendinitis	12 (31.5)
Suprapsinatus tendinitis/rupture	21 (55.2)
Bursitis	23 (60.5)
ACJ degeneration	20 (53)

Quick-DASH: Quick disabilities of the arm, shoulder and hand; FIM: Functional independence measures; SF-36: short form-36; ACJ: acromioclavicular joint

**Table 3:** Multivariate analyses of outcomes associated with upper extremity overuse syndromes.

Variables	n (%)	B	OR (95% CI)	p
**CTS**	8 (21)			
Pain in hand		4.85	128 (1.91–8415)	**0.023**
**CuTS**	2 (5.2)			
Ulnar neuropathy-USG		1.12	3.06 (1.19–7.97)	**0.025**
**Lateral epicondylitis**	6 (15.8)			
Pain in elbow		4.16	64.6 (3.15–1325)	**0.007**
Pain with wrist extention		3.49	33.0 (1.28–851)	**0.035**
**Medial epicondylitis**	2 (5.2)			
Medial epicondylitis-USG		3.06	21.3 (1.47–310)	**0.025**
**Biceps tendinitis**	4 (10.5)			
Pain in shoulder		3.21	25.0 (1.52–411)	**0.024**
SF-36 **(**Physical functioning)		−0.03	0.962 (0.93–0.999)	**0.043**
**Rotator cuff tendinitis/rupture**	12 (31.6)			
Pain in shoulder		4.75	116 (5.55–2429)	**0.002**
SF-36 (Emotional health)		0.10	1.11 (1.01–1.22)	**0.024**
**Subacromial bursitis**	12 (31.6)			
Pain in shoulder		3.87	3.62 (3.62–643)	**0.003**
Group 1 tests		4.29	73.5 (3.80–1423)	**0.004**
**ACJ degeneration**	10 (26.4)	-	-	**-**

CTS: carpal tunnel syndrome; CuTS: cubital tunnel syndrome; SF-36: short form-36; USG: ultrasonography; ACJ: acromioclavicular joint; OR: Odds ratio

**Table 4:** Multivariate analysis of patients with and without overuse syndromes.

Independent variables	B	OR (95% CI)	p
**Wheelchair use** (hour/day)	0.51	1.67(1.004–2.77)	0.048
**SF-36 (Pain)**	−0.059	0.94(0.89–0.99)	0.027
**Level of injury**	2.48	12.0(0.97–148)	0.052

SF-36: short form-36; OR: Odds ratio.
